# Acute Limb Ischemia as the Initial Severe Presentation in COVID-19

**DOI:** 10.7759/cureus.14226

**Published:** 2021-03-31

**Authors:** George Horani, Kunwar Kaur, Prem Patel, Hartaj Virk, Fayez Shamoon

**Affiliations:** 1 Internal Medicine, St. Joseph's University Medical Center, Paterson, USA; 2 Cardiology, St. Joseph's University Medical Center, Paterson, USA

**Keywords:** covid-19, acute limb ischemia, thromboembolism, hypercoagulable state

## Abstract

Coronavirus disease 2019 (COVID-19), caused by the novel severe acute respiratory syndrome coronavirus 2 (SARS-CoV-2), is characterized by an overwhelming inflammatory response in a subset of patients, resulting in respiratory compromise, multiorgan failure, and death. A common complication seen in patients hospitalized with COVID-19 infections is the development of venous and arterial thromboemboli. This occurs especially in patients who suffer from severe respiratory or systemic manifestations on the COVID-19 spectrum of disease. We present a case of acute limb ischemia as the initial presentation in a patient who tested positive for COVID-19.

## Introduction

With more than 20 million cases and over 400,000 deaths in the United States since January 2020 [1], coronavirus disease 2019 (COVID-19) caused by the novel severe acute respiratory syndrome coronavirus 2 (SARS-CoV-2) continues to overwhelm the medical community. Since its early appearance, COVID-19 has manifested as a respiratory illness, with symptoms ranging from mild cough and congestion to severe, life-threatening respiratory failure. In addition to respiratory symptoms, widespread cases of thromboembolism, both venous and arterial, have been reported in patients hospitalized with COVID-19 infections. The proposed mechanism of thrombus formation in these patients is multifactorial and involves the virus affecting the platelets and endothelium leading to a coagulopathic state and thromboembolism [2]. Recent updates from the American Society of Hematology in January 2021 recommend the use of prophylactic anticoagulation in all adults hospitalized with COVID-19 [3]. Patients in the US have been cautioned by the Centers for Disease Control and Prevention (CDC) to look out for symptoms such as fever, chills, or shortness of breath and to seek medical attention if they develop difficulty breathing, chest pain, or any blue or gray skin discoloration signifying hypoxia. These warnings do not include routine surveillance for symptoms patients may experience with thromboembolic complications of COVID-19 [4]. We report an incident of acute limb ischemia (ALI) secondary to COVID-19, which presented prior to respiratory symptoms and unfortunately failed medical and surgical intervention, complicating the patient’s clinical course. 

## Case presentation

A 60-year-old male with a medical history of diabetes mellitus type II, dyslipidemia, and a diagnosis of COVID-19 infection two weeks prior presented complaining of a cold right foot for four days. His initial symptoms consisted of mild fever, nausea, vomiting, and diarrhea which started at the time of his COVID-19 diagnosis. In the week prior to presentation, he developed a dry cough and completed five days of azithromycin, prescribed by his primary care physician. However, on the day of presentation to the hospital, he had new onset shortness of breath which prompted his visit. On arrival, he was afebrile with a temperature of 36.7 degrees Celsius, tachypneic at a rate of 30 breaths per minute, saturating 55% on ambient room air. D-dimer, international normalized ratio (INR), and lactic acid were elevated at >20mcg/mL (reference range <0.5mcg/mL), 1.5, and 4.8mmol/L (reference range 0.5 - 2.2mmol/L) respectively. During the first week of hospitalization, all of these values trended down to normal reference range levels. Chest x-ray showed peripheral pulmonary opacities and consolidations bilaterally suspicious for atypical viral pneumonia. Electrocardiogram showed normal sinus rhythm with normal intervals without any signs of ischemia. 

On physical examination, the right foot was cool to touch with diminished distal pulses. Given these findings, the patient was immediately started on a therapeutic dose of enoxaparin. In addition, as part of COVID-19 management, he was given dexamethasone, remdesivir, and tocilizumab, along with supplemental oxygen via high flow nasal cannula and subsequently bilevel positive airway pressure. However, the patient remained hypoxic and was intubated and placed on mechanical ventilation on the same day.

**Figure 1:**
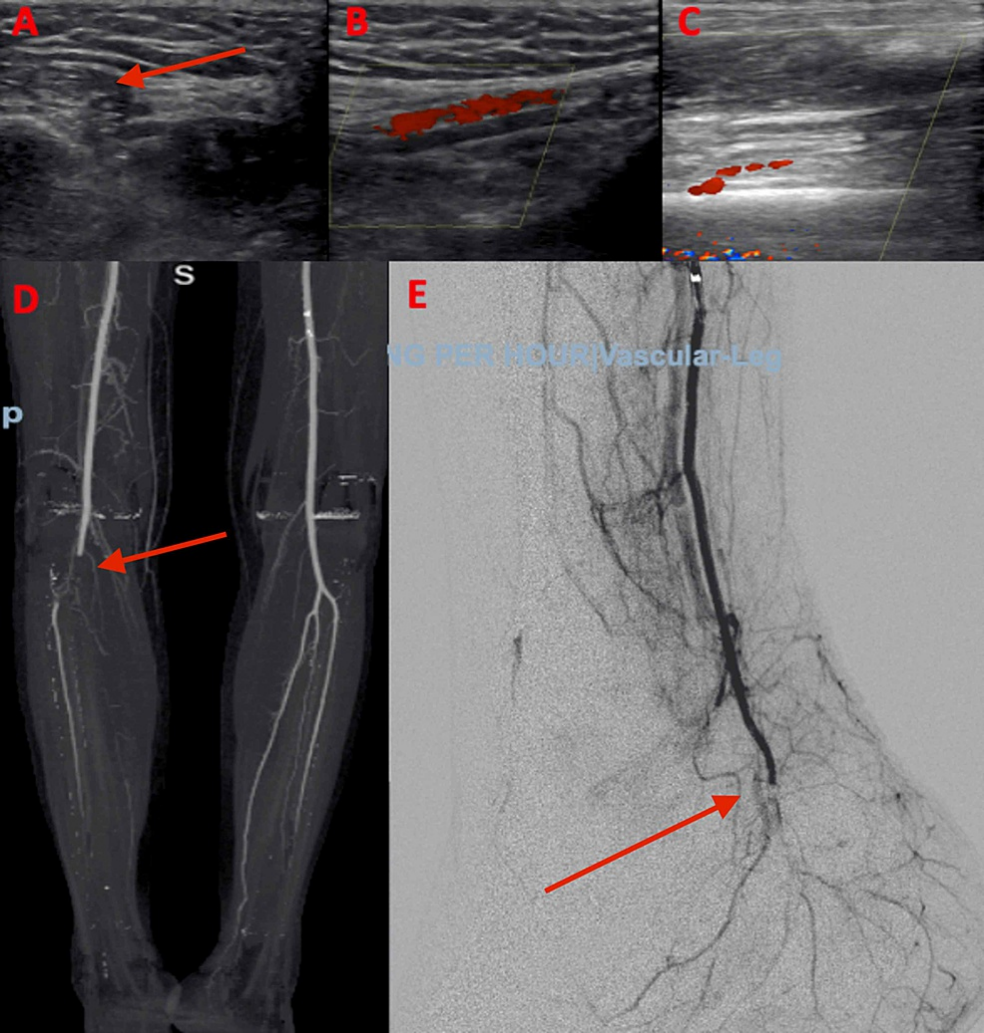
Arterial occlusion in COVID-19 infection. A-B: Arterial duplex of the right posterior tibialis showing the clot with no distal flow. C: Arterial duplex of the right dorsalis pedis with occlusion. D: CT angiography showing occlusion at the junction of the popliteal and peroneal posterior tibial trunk. E: Angiogram showing no flow to the distal right foot.

An arterial duplex ultrasound of the right leg was performed, revealing significant stenosis below the knee (Figure 1A-1C). Subsequent CT angiography showed total occlusion at the junction of the popliteal and peroneal posterior tibial trunk (Figure 1D). A right leg angiogram was then performed and revealed 100% occlusion into the trifurcation of the distal part of the right popliteal artery; the right anterior tibial (AT), posterior tibial (PT), and peroneal arteries were 100% occluded as well (Figure 1E). He underwent thrombolysis and thrombectomy of the AT and PT arteries followed by catheter-based thrombolysis for 12 hours. Despite this treatment, the foot remained cold and mottled with no pulses. Vascular surgery was consulted, and the patient went for balloon angioplasty and mechanical thrombectomy of the superficial femoral artery, AT artery, PT artery, and dorsalis pedis (DP) arch. This resulted in return of Doppler-identified PT pulse only, but a follow-up duplex arterial study showed persistent occlusive disease in the remaining blocked arteries. Amputation was planned however the patient became hemodynamically unstable and eventually succumbed to COVID-19-related acute respiratory distress syndrome and cardiopulmonary failure. 

## Discussion

The novel SARS-CoV-2 virus has been shown to affect the respiratory system, with afflicted individuals being either asymptomatic, demonstrating mild upper respiratory symptoms, or developing respiratory failure requiring noninvasive and invasive ventilation. Other systemic symptoms, including fevers, chills, and extrapulmonary symptoms such as headaches, myalgias, and diarrhea have also been reported [5]. In addition, the first case of COVID-19 presenting with cerebral venous thrombosis was reported as early as May 2020 [6]. Since then, many other cases of thromboembolism have been described in relation to COVID-19 leading to ischemic strokes and pulmonary emboli [7,8].

In addition to strokes and pulmonary emboli, acute limb ischemia (ALI) secondary to COVID-19 has been described in the literature as early as April 2020. Case reports and studies have demonstrated not only the development of ALI despite prophylactic anticoagulation but also lower rates of successful revascularization [9-11]. Generally, and for the exception of graft thrombosis, causes of ALI are either arterial embolism (30-46%) or thrombosis (20-40%). Cardiac or aortic embolization, hypercoagulable states, and iatrogenic complications from endovascular procedures are also associated with a decrease in limb perfusion leading to ALI [12].

The pathophysiology of thromboembolic phenomena in COVID-19 is multifactorial. As discussed by Zakeri et al., monocyte activation secondary to viral infection releases a proinflammatory cytokine storm which in turn results in neutrophil recruitment and tissue factor activation, binding factor VII and activating the coagulation pathway [13]. Another described mechanism leading to the dysfunctional circulatory changes is endeliopathy [13,14]. This mechanism proposes that after viral adhesion to the angiotensin converting enzyme (ACE) receptors on the endothelial cells, the virus replicates and infiltrates the cell causing inflammation and endothelial cell apoptosis, which in turn promotes a hypercoagulable state [14]. Furthermore, the infection causes the release of von Willebrand factor (VWF), which plays a role in platelet activation and thrombus formation [13]. We currently have recommendations supporting the use of anticoagulation for thromboprophylaxis and treatment of thromboembolism in COVID-19; the use of low molecular weight heparin or unfractionated heparin has been suggested [3,15,16]. On the contrary, use of anticoagulant and antiplatelet agents for prevention of venous or arterial thrombosis in COVID-19 patients treated in the outpatient setting has not been recommended [17]. 

Since the emergence of COVID-19, it has been recognized and widely accepted as a respiratory virus, primarily causing mild constitutional and lower respiratory tract symptoms. Most patients that are hospitalized with COVID-19 infections, therefore, complain of shortness of breath and are typically hypoxic on presentation. Interestingly, ALI was the initial serious presentation in this case, unbeknown to the patient who did not seek medical attention for his cold foot, which began several days prior to his shortness of breath. This highlights the importance of perhaps educating patients not only on the respiratory symptoms that accompany COVID-19 infections, but also the thromboembolic complications that may present with skin changes, extremity swelling, and pain. 

## Conclusions

COVID-19 infection predisposes to arterial and venous thrombi by inducing a hypercoagulable state. This has been explained by the viral effect on the platelets and endothelium leading to inflammation and eventually thrombus formation. Despite prophylactic use of anticoagulation, the prothrombotic effects of the infection continue to burden the cardiovascular system. Furthermore, studies have shown that despite intervention, success rates of revascularization in COVID-19 patients are lower than in noninfected patients. Finally, it is important to recognize thromboembolic events in patients with COVID-19 infections as it complicates the recovery process and leads to poor clinical outcomes. 
